# Supplementary material for: CRISPR Gene Editing of Murine Blood Stem and Progenitor Cells Induces MLL-AF9 Chromosomal Translocation and MLL-AF9 Leukaemogenesis
Source: Int J Mol Sci. 2020 Jun 15;21(12):4266. doi: 10.3390/ijms21124266 (PMC7352880; doi:10.3390/ijms21124266)
Supplement: Supplementary file 1 [file ijms-21-04266-s001.zip › Supplementary/Keeshan-original gels.docx]

**CRISPR Gene Editing of Murine Blood Stem and Progenitor Cells Induces MLL-AF9 Chromosomal Translocation and MLL-AF9 Leukaemogenesis**

Evgenia Sarrou ^1^, Laura Richmond ^1^, Ruaidhrí J. Carmody ^2^, Brenda Gibson ^3^ and Karen Keeshan ^1,^*

## **Original Gels**

### *Surveyor Assay*


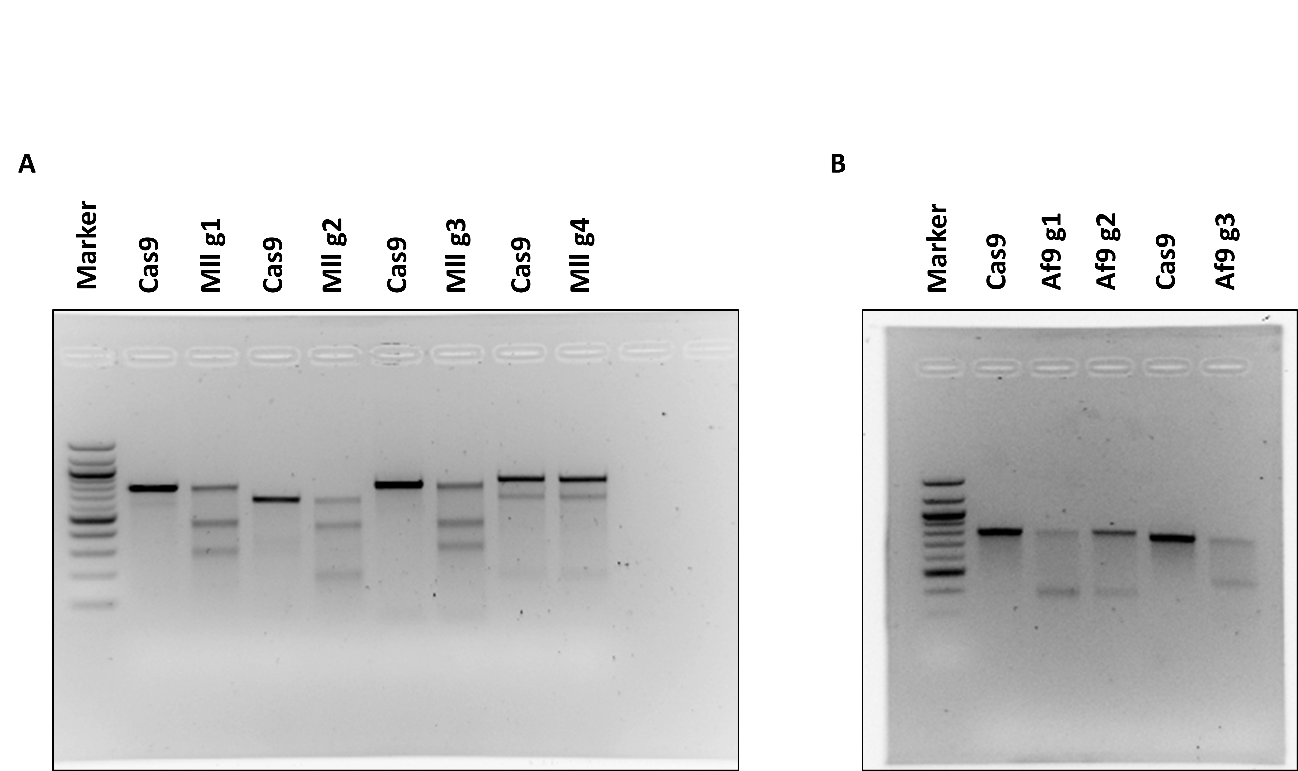


**Figure S2.** Uncropped gel electrophoresis from figure 1C, of Surveyor products from Mll (**A**) and Af9 (**B**) single guides. Guides are shown against New England Biolabs Quickload 100bp DNA Ladder.

### **Verification of translocation**


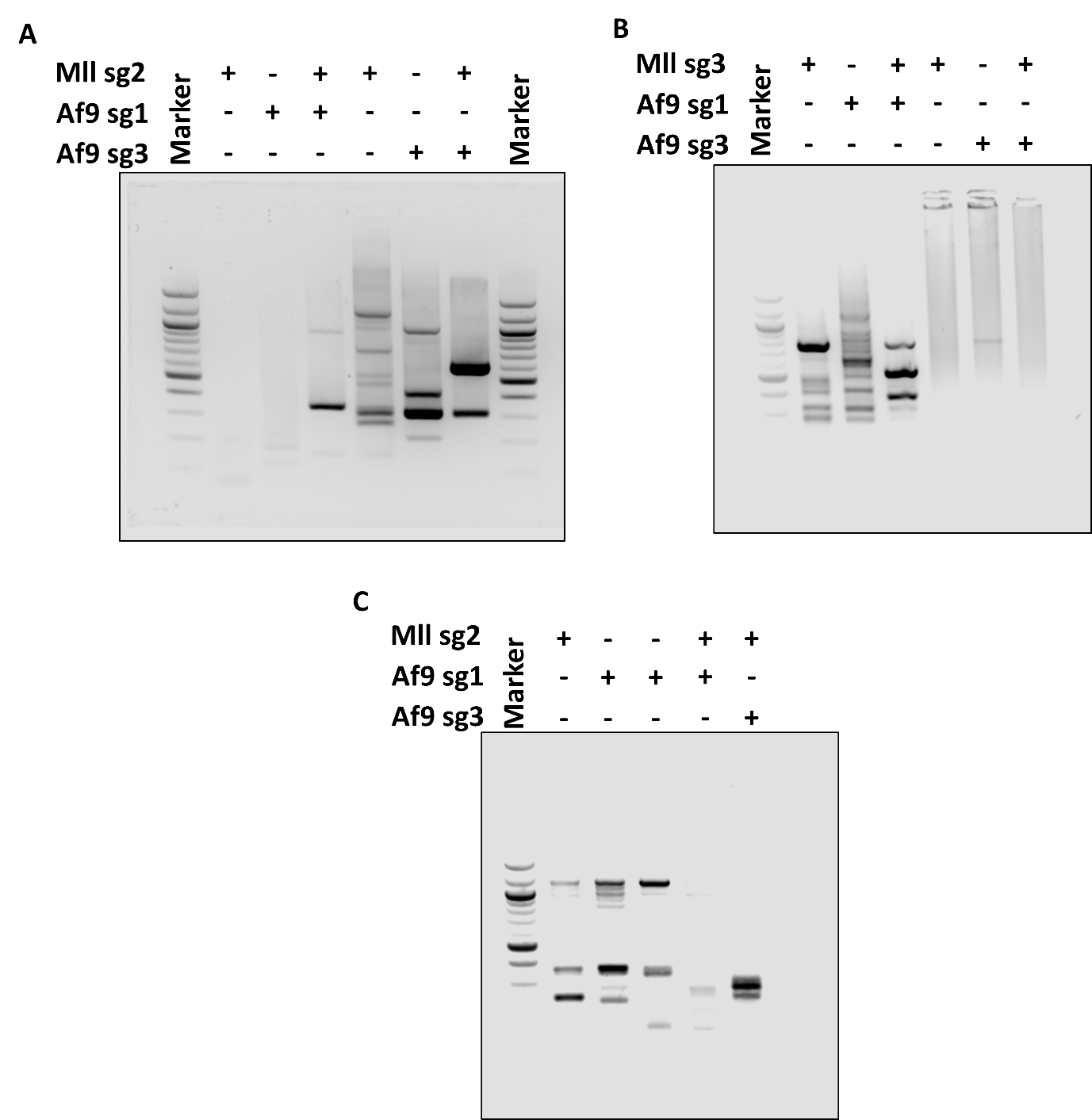


**Figure S3.** Uncropped gel electrophoresis from figure 2C and 2E of amplicons from (**A–B**) genomic DNA PCR and (**C**) RT-PCR. Marker is New England Biolabs Quickload 100bp DNA Ladder.
