# Supplementary material for: CRISPR Gene Editing of Murine Blood Stem and Progenitor Cells Induces MLL-AF9 Chromosomal Translocation and MLL-AF9 Leukaemogenesis
Source: Int J Mol Sci. 2020 Jun 15;21(12):4266. doi: 10.3390/ijms21124266 (PMC7352880; doi:10.3390/ijms21124266)
Supplement: Supplementary file 1 [file ijms-21-04266-s001.zip › Supplementary/Keeshan-supplementary.docx]

**CRISPR Gene Editing of Murine Blood Stem and Progenitor Cells Induces MLL-AF9 Chromosomal Translocation and MLL-AF9 Leukaemogenesis**

Evgenia Sarrou ^1^, Laura Richmond ^1^, Ruaidhrí J. Carmody ^2^, Brenda Gibson ^3^ and Karen Keeshan ^1,^*

## **Supplementary Methods**

### *sgRNA synthesis*

Oligos were phosphorylated using 10 units of T4 polynucleotide kinase (PNK) and duplexed then diluted 1:200 before ligation into Esp3I-digested pL.CRISPR.EFS.GFP. PlasmidSafe exonuclease (Epicentre) was used to remove non-ligated DNA before transformation into One Shot Stbl3 E. Col (Invitrogen) and colonies were growth on LB agar supplemented with 1ug/ml Ampicillin. Individual colonies were prepared by QIAprep mini or midi prep (QIAgen) and sequence validated.

**Table S1.** ssDNA oligo sequences of sgRNAs.

| Target gene | ID | Top oligo (5’-3 ’) | Bottom oligo (5’-3 ’) |
| --- | --- | --- | --- |
| *Mll* | Mll­ sg1 | CACCGCACTCTGTACGTCAA GC | AAACGCTTGACGTACAGA GTGC |
|  | Mll sg2 | CACCGTGTAAGCTTTCGCCC CTCT | AAACAGAGGGGCGAAAGC TTACAC |
|  | Mll sg3 | CACCGTACAGTGAACCACCG GATGT | AAACACATCCGGTGGTTC ACTGTAC |
|  | Mll sg4 | CACCGCGTCAGATCTCATTA CGGG | AAACCCCGTAATGAGATCT GACGC |
| *Af9* | Af9 sg1 | CACCGTTAGCCCCCTGCCG GTTGT | AAACACAACCGGCAGGGG GCTAAC |
|  | Af9 sg2 | CACCGTTATCCCTCCAACAA CCGGC | AAACGCCGGTTGTTGGAG GGATAAC |
|  | Af9 sg3 | CACCGGTGGCCATCCGCTA GTTG | AAACCAACTAGCGGATGG CCACC |

###

**Figure S1**. pL-CRISPR.EFS.GFP map with relevant sequence features indicated

### *Antibodies and flow cytometry reagents*

**Table S2.** Antibodies and flow cytometry stains.

| Target | ID | Supplier |
| --- | --- | --- |
| c-Kit (CD117) | 2B8 | eBioscience |
| DAPI | N/A | Sigma |
| CD11b | M1/70 | eBioscience |
| Gr1 (Ly-6G) | RB6-8CS | eBioscience |
| F4/80 | BM8 | eBioscience |

### *Surveyor*

Genomic DNA was PCR amplified with Hercules II fusion polymerase (Agilent) using primers flanking the Cas9 cleavage site of each sgRNA (**Table S3**). PCR products were purified using the QIAQuick PCR purification kit (QIAgen) and used for heteroduplex formation before digestion with Surveyor nuclease S. Reactions were visualised on a 2% agarose gel and analysed using ImageJ software to calculate indel percentage.

**Table S3.** PCR primers for amplification of genomic DNA

| Target Gene | Target Site | Forward Primer (5’-3 ’) | Reverse Primer (5’-3 ’) |
| --- | --- | --- | --- |
| *Mll* | sg1 | TAATGGATCCCCCTCTGGTTGATTGGTGT | TAATCTAGATACCTAACCTGCCTGTTAGC |
|  | sg2 | TAAGAATTCCCCGCCAAGTATCCCTGTAAA | TAATCTAGACAAGAGGGGAGAATACAAATGCC |
|  | sg3 | TAAGGATCCTGAGTAAGCAGATTTGGAGGGG | TAATCTAGAAGGCAGAAACAGAAAAAGGACCTC |
|  | sg4 | TAAGAATTCTCCCCATAACACCCAGAGTAGT | TAAGAAGCTTGGCTAATGACCATCCCACCATA |
| *Af9* | sg1/3 | TAAGGGATCCCTTTGCACGCAGCAAGATTC | TAACTCTAGAAACTCAATGCACAATGGGACTTTT |
|  | sg2 | TAACGGATCCCTCAGCCTTTTCAAGCTATGGG | TAATCTAGAGCTACCCATATAAACAGGCACTC |

### *Reverse Transcription PCR*

**Table S4.** Primers for RT-PCR

| Target Gene | Target Site | Forward Primer (5’-3 ’) | Reverse Primer (5’-3 ’) |
| --- | --- | --- | --- |
| *Mll* | Exon 8/9 | TAAGAATTCCGTTCCAAGTGAGCCCAAGA | TAAGGATCCTAAGCCTCCCATCTCCCACA |
|  | Exon 10/11 | TATGGTACCAGAAGCCACCTCCAGTAAGT | TATGGATCCGTGGGTTTGGTGGGGTAGTTT |
| *Af9* | Exon 8/9 | TAAGGTACCTGATGACAACGAGGAGGAGTC | TATAAGCTTGCAGAATGTGTCGTTCCCTCA |

### *qRT-PCR*

**Table S5.** qPCR primer sequences.

| Target | Forward Primer (5’-3 ’) | Reverse Primer (5’-3 ’) |
| --- | --- | --- |
| *Gusb* | GGGACAAAAATCACCCTGCG | GCGTTGCTCACAAAGGTCAC |
| *Hprt1* | GAGAGCGTTGGGCTTACCTC | ATCGCTAATCACGACGCTGG |
| *Hoxa9* | CCCTGACTGACTATGCTTGTGGT | TCTCCGCCGCTCTCATTCTC |
| *Cdk6* | AGAAGTCCTGCTCCAGTCCA | CACGTCTGAACTTCCACGAA |
| *Dot1l* | GGAACCGTTGGAGGTAATTAGGA | ATTCACAGTGGCTCCATGTCC |
| *Sirt1* | CTGAAAGTGAGACCAGTAGCA | GATGAGGCAAAGGTTCCCTA |
| *Suv39h1* | TTACAACATGCAAGTGGACCC | GGTATTTTCGGCAAGCCGTT |

## **Supplementary data**

### *Surveyor Assay*


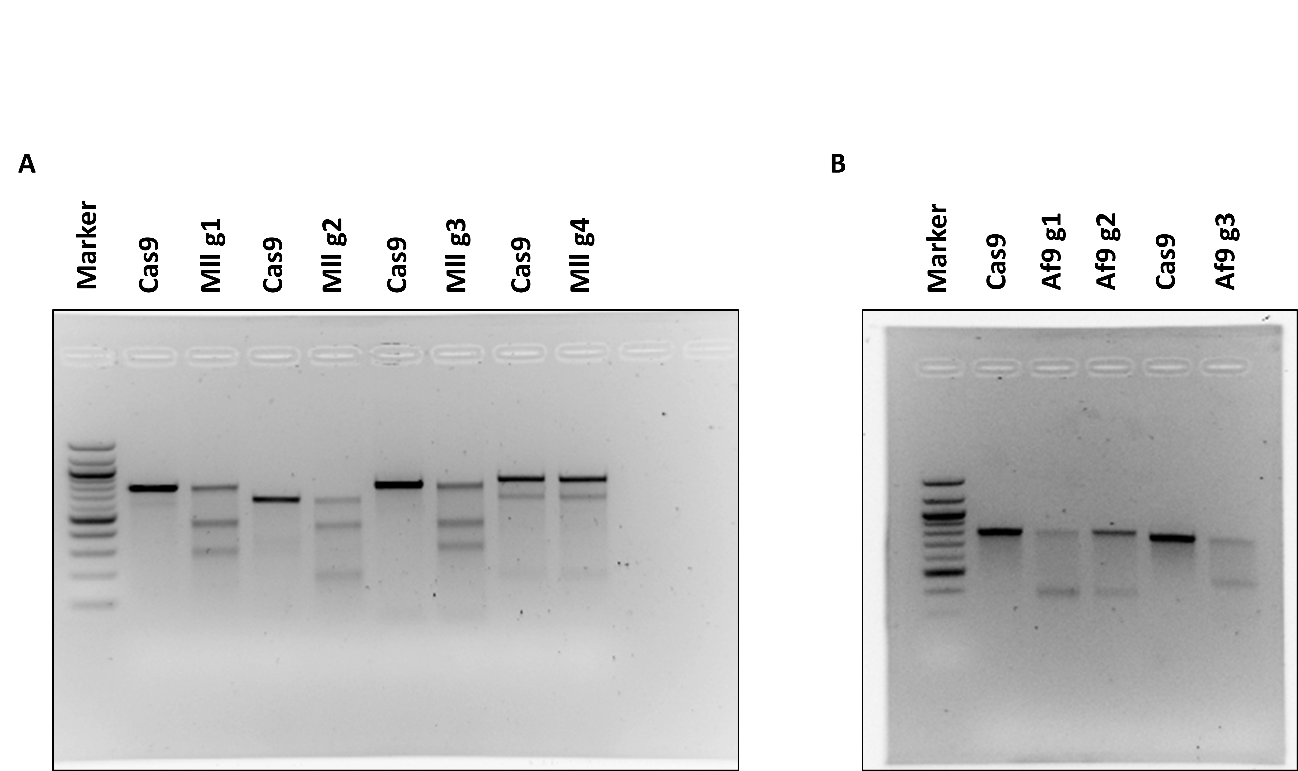


**Figure S2.** Uncropped gel electrophoresis from figure 1C, of Surveyor products from Mll (**A**) and Af9 (**B**) single guides. Guides are shown against New England Biolabs Quickload 100bp DNA Ladder.

### **Verification of translocation**


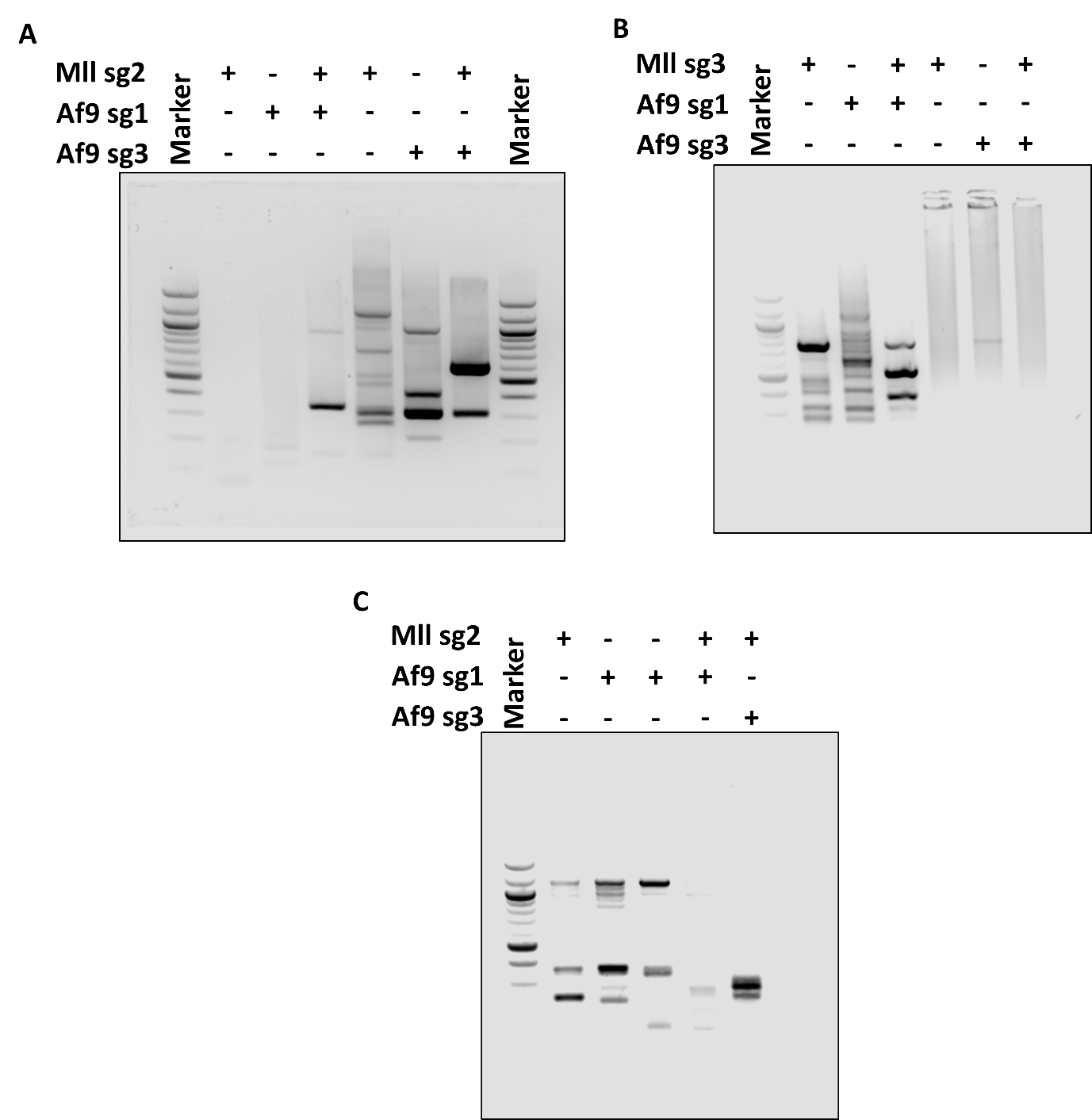


**Figure S3.** Uncropped gel electrophoresis from figure 2C and 2E of amplicons from (**A–B**) genomic DNA PCR and (**C**) RT-PCR. Marker is New England Biolabs Quickload 100bp DNA Ladder.
